# Supplementary material for: The temporal structure of the inner retina at a single glance
Source: Sci Rep. 2020 Mar 10;10:4399. doi: 10.1038/s41598-020-60214-z (PMC7064538; doi:10.1038/s41598-020-60214-z)
Supplement: Supplementary file 1 — Supplementary information. [file 41598_2020_60214_MOESM1_ESM.pdf]

The temporal structure of the inner retina at a single glance

Zhijian Zhao[1,2]*, David Klindt[1-5]*, André Maia Chagas[1,2,4], Klaudia P. Szatko[1,3,4], Luke Rogerson[1-4], Dario A. Protti[7], Christian Behrens[1-3], Deniz Dalkara[8], Timm Schubert[1,2], Matthias Bethge[2,3,5,9], Katrin Franke[1,3#], Philipp Berens[1-3,6#], Alexander Ecker[2,3,5,9#], Thomas Euler[1-3#]

Supplementary Figure(s)

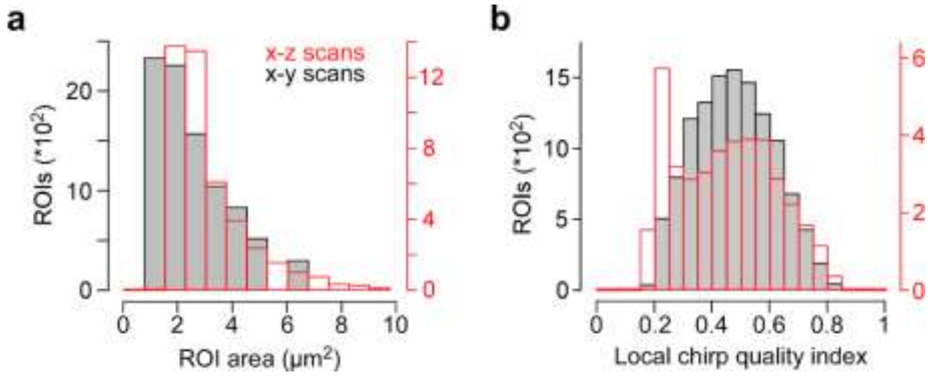

***Supplementary Figure S1** | ROI sizes and local chirp quality indices in x-z and x-y scans of the IPL.*
***a**, Distribution of ROI areas for all ROIs in x-z (red) and x-y (black) scans. x-y scan data from* ref[1].
***b**, Distribution of local chirp quality index for x-z (red) and x-y (black) scans.*

1. Franke, K. *et al.* Inhibition decorrelates visual feature representations in the inner retina. *Nature* **542**, (2017).
